# Beyond Universal Coverage: The Magnitude of the Oral Health Equity Gap Between Socially Excluded Children and a Reference Paediatric Population Within a Single Spanish Setting

**DOI:** 10.3390/children13070919

**Published:** 2026-07-12

**Authors:** Mónica Fernández-Mafé, Lucía Miralles-Jordá, Julián Espinosa-Giménez, Anna Paradowska-Stolarz, María Dolores Gómez-Adrián, María Ester Legidos-García, María Teresa Murillo-Llorente, Marcelino Pérez-Bermejo

**Affiliations:** 1 Oral Surgery Unit, Department of Dentistry, School of Medicine and Health Sciences, Catholic University of Valencia, 46007 Valencia, Spain; monica.fernandez@ucv.es (M.F.-M.); lucia.miralles@ucv.es (L.M.-J.); julian.espinosa@ucv.es (J.E.-G.); mariadolores.gomez@ucv.es (M.D.G.-A.); 2 Division of Dentofacial Anomalies, Department of Orthodontics and Dentofacial Orthopedics, Wroclaw Medical University, Krakowska 26, 50-425 Wroclaw, Poland; anna.paradowska-stolarz@umw.edu.pl; 3 SONEV Research Group, School of Medicine and Health Sciences, Catholic University of Valencia, 46007 Valencia, Spain; ester.legidos@ucv.es (M.E.L.-G.); mt.murillo@ucv.es (M.T.M.-L.)

**Keywords:** dental caries, oral health inequalities, social determinants of health, children, social exclusion, universal health coverage, equity gap

## Abstract

**Highlights:**

**What are the main findings?**

- Socially vulnerable children attending schools serving communities at a high risk of social exclusion (“escuelas singulares”; *colegios de acción educativa singular*) showed a substantially higher burden of dental caries than a reference paediatric population from the same Spanish urban setting.
- The oral health gap persisted after adjustment for age, sex, toothbrushing frequency and sugar intake, suggesting that the measured individual behaviours alone are unlikely to explain the observed inequalities.

**What are the implications of the main findings?**

- Universal health coverage does not necessarily guarantee equitable oral health outcomes when access to preventive and restorative dental care remains socially patterned.
- School-based, targeted preventive and restorative strategies should be integrated into universal oral health policies to reduce inequalities among socially excluded children.

**Abstract:**

Background/Objectives: Dental caries is socially patterned, but the magnitude of the inequality associated with social exclusion is difficult to quantify when populations are compared across different countries and health systems. This study estimated the oral health gap between socially vulnerable children attending escuelas singulares and a contemporaneous reference paediatric population attending university dental clinics within the same city and health-system context. Methods: A cross-sectional analytical study was conducted in 231 children aged 6–12 years in Valencia, Spain: 129 from two state-funded schools serving communities at high risk of social exclusion, hereafter referred to as escuelas singulares (Colegio Madre Petra, *n* = 64; Colegio Santiago Apóstol, *n* = 65), and 102 reference children examined at the University Dental Clinics of the Catholic University of Valencia. Caries experience (DMFT/dmft-derived global score), the Simplified Oral Hygiene Index (OHI-S), toothbrushing frequency and sugar consumption were recorded under standardised conditions. The groups were compared with non-parametric tests, and multivariable logistic and modified Poisson regression models estimated the independent association (adjusted OR and adjusted prevalence ratio, respectively) between school context and the presence of caries, adjusting for age, sex and behaviours. Results: Caries experience was markedly higher in vulnerable children (global caries score 4.07 ± 3.44 vs. 0.61 ± 1.96; *p* < 0.001), with a caries prevalence of 76.0% vs. 13.7% (a prevalence ratio of 5.5) and a 6.7-fold higher mean burden. Only 24.0% of vulnerable children were caries-free versus 86.3% of reference children. Vulnerable children also showed poorer oral hygiene (OHI-S 1.49 ± 0.84 vs. 0.19 ± 0.34; *p* < 0.001), less frequent brushing (50.4% vs. 79.4% brushing ≥ twice daily; *p* = 0.002) and higher sugar consumption (*p* < 0.001). After adjustment for age, sex, brushing and sugar, attendance at an escuela singular was associated with an adjusted prevalence ratio of 5.4 (95% CI 3.3–8.9; primary effect measure) and, in a secondary logistic model, with an adjusted OR of 20.5 (95% CI 9.6–43.8). Conclusions: Within a single high-income setting with formally universal health coverage, social exclusion was associated with a very large, graded excess of childhood caries that the measured behavioural differences alone were unlikely to explain, underscoring the role of social and structural determinants beyond individual behaviours. These cross-sectional findings are consistent with proportionate-universalism strategies (targeted, school-based preventive and restorative care embedded within the universal system), since formal coverage alone may be insufficient to achieve oral health equity.

## 1. Introduction

Dental caries is the most prevalent non-communicable disease of childhood and a paradigmatic example of a socially patterned condition. Although its biological mechanism (demineralisation of dental hard tissues by acids derived from the bacterial fermentation of dietary sugars) is well established [1,2], its distribution in populations is governed largely by social, commercial and structural determinants rather than by individual biology alone [3,4]. The Lancet Series on Oral Health reframed caries as a disease embedded in the wider social and commercial environment, calling for a decisive shift away from approaches that place responsibility solely on individual behaviour [3,4].

A consistent socioeconomic gradient in caries has been documented across high-, middle- and low-income countries: children of lower socioeconomic position experience more caries, more untreated decay and poorer access to care [5,6]. The resulting burden of untreated disease remains substantial worldwide [7,8,9]. In Spain, regional surveillance in the Valencia Community reported a caries prevalence of approximately 37% in the deciduous dentition of 6 year olds and 30% in the permanent dentition of 12 year olds [10], figures that provide a useful population-level benchmark against which the present findings can be interpreted. Crucially, this gradient can persist even where health coverage is, in principle, universal. Spain operates a tax-funded National Health System with near-universal coverage, yet oral health care is only partly embedded within publicly funded health-care arrangements, leaving room for marked inequalities among the most disadvantaged groups [10,11]. These inequalities may operate through multiple pathways, including delayed or episodic use of dental services, lower oral-health literacy, reduced capacity to maintain preventive routines, competing family priorities, material deprivation and unequal exposure to protective resources such as fluoride and timely restorative care.

Quantifying the magnitude of social inequality in oral health is methodologically demanding. Comparisons between countries or regions are confounded by differences in living standards, fluoride exposure, dietary culture, workforce density and the organisation of dental services, so that the observed gap reflects an entangled mixture of social position and health-system context. A more informative design holds the system context constant and contrasts a socially excluded group with a reference group drawn from the same city, the same period and the same health-system environment. The difference that remains can then be interpreted with less cross-system confounding, although causal attribution remains limited in a cross-sectional study.

In the Spanish education system, schools serving communities at high risk of social exclusion (“escuelas singulares”; *colegios de acción educativa singular)* are publicly funded institutions serving children and families exposed to socioeconomic disadvantage, migration-related vulnerability or multidimensional poverty. Earlier work in these same schools reported a substantially elevated caries burden [12], consistent with regional data showing markedly lower caries levels in the general child population [11,13,14]. They therefore offer access to a paediatric population that is socially vulnerable yet covered by the same broad public system as the general population. A pragmatic reference group is provided by children who attend university dental clinics for routine paediatric dental care; this group should be interpreted as a non-excluded clinical comparator from the same urban setting, rather than as a fully population-based sample.

The present study was designed to measure the oral health equity gap within a single high-income setting by comparing caries experience, oral hygiene, toothbrushing and sugar consumption between children attending two escuelas singulares and a contemporaneous reference paediatric population attending the University Dental Clinics of the Catholic University of Valencia. By holding country, city and health system broadly constant, the study provides a within-system estimate of the association between social exclusion and childhood caries and explores how much of the resulting gap can (and cannot) be explained by individual behaviours. This perspective is consistent with broader evidence showing that disparities in access to oral health care and structural barriers strongly shape oral-health inequalities [15].

## 2. Materials and Methods

### 2.1. Study Design

This was a cross-sectional, observational and analytical study conducted in Valencia, Spain, from October 2025 to November 2025. Reporting followed the Strengthening the Reporting of Observational Studies in Epidemiology (STROBE) guidelines for cross-sectional studies [16].

### 2.2. Study Population and Setting

A total of 231 children aged 6–12 years were studied across two groups recruited in the same city and period. The vulnerable group comprised 129 children from two state-funded schools serving communities at high risk of social exclusion, referred to hereafter as escuelas singulares: Colegio Madre Petra (*n* = 64) and Colegio Santiago Apóstol (*n* = 65). The reference group comprised 102 healthy children who were present at the University Dental Clinics of the Catholic University of Valencia (UCV) as accompanying children of patients receiving dental care. They were not recruited because of a dental complaint or treatment need and were included only after parental or legal guardian informed consent had been obtained. This group served as a non-excluded comparator from the same urban setting. Both groups were drawn from the same metropolitan area and were therefore exposed to the same national health system, the same regional dental-care arrangements and the same broad environmental conditions, including water-supply characteristics. The sampling strategy was non-probabilistic and pragmatic: children were recruited from the two participating escuelas singulares and from accompanying children present at the university dental clinics during the study period, provided that eligibility criteria were met and parental or legal guardian consent was obtained.

### 2.3. Inclusion and Exclusion Criteria

Children aged 6–12 years with signed informed consent from a parent or legal guardian and able to cooperate with an oral examination were included. Children with systemic conditions affecting dental development, those unable to cooperate and those with incomplete data for the primary study variables were excluded. Children with developmental structural defects of the tooth surface (e.g., amelogenesis/dentinogenesis imperfecta or severe molar-incisor hypomineralisation) that could impair accurate caries scoring were also excluded.

### 2.4. Variables and Measurement Instruments

Caries experience was assessed using the WHO-standard DMFT index for permanent dentition (decayed, missing due to caries and filled teeth; Spanish equivalent: CAOD) and the corresponding lower-case dmft index for primary teeth (Spanish equivalent: cod). Given the mixed dentition of the 6–12-year age range, a global caries score was also calculated as DMFT + dmft, with no tooth double-counted across dentitions, and used as a mixed-dentition summary measure. DMFT and dmft are additionally reported separately by group in Section 3.2 for comparability with WHO-standard reporting. Examinations followed standardised WHO criteria [17], performed by calibrated examiners under artificial light using sterile plane mouth mirrors and WHO periodontal probes; no radiographs were taken.

Oral hygiene was assessed with the Simplified Oral Hygiene Index (OHI-S) [18], based on debris and calculus on index tooth surfaces, and categorised as good (0.0–0.6), fair (0.7–1.8) or poor (1.9–3.0). Toothbrushing frequency was self-reported and coded as never (0), once daily (1), twice daily (2) or three or more times daily (3+). Sugar consumption was assessed with a structured dietary questionnaire developed for this study and dichotomised as lower (≤once daily) or higher (>once daily) frequency of intake. This instrument was not formally validated and captured frequency rather than amount, timing, food matrix or total free-sugar exposure. Age (years) and sex (1 = male, 2 = female) were recorded as sociodemographic variables. Individual-level socioeconomic variables, including parental education, household income, parental occupation and migration background, were not collected.

### 2.5. Data Collection Procedures

Data were collected on a standardised clinical record form. Examiners underwent joint training and calibration before data collection; inter-examiner reliability (Cohen’s kappa) exceeded 0.85 for the main clinical variables. School examinations were performed in an upright position under an LED headlamp; clinic examinations followed equivalent standardised protocols. The same pool of calibrated examiners assessed children in both settings, but it was necessary for examiners to be aware of the examination location and therefore they were not blinded to group/exposure status; the reported kappa values reflect inter-rater agreement on diagnostic criteria and do not rule out differential detection related to setting-specific examination conditions (upright/LED-headlamp in schools versus standard clinic conditions), which is acknowledged as a potential source of bias in Section 4.6. Behavioural data were obtained through structured interviews with children, supplemented by parental reports where available.

### 2.6. Statistical Analysis

Analyses were performed in SPSS v26.0 (IBM Corp., Armonk, NY, USA), with the statistical significance set at *p* < 0.05 and 95% confidence intervals reported. Continuous variables were summarised as mean ± standard deviation and as median (interquartile range, IQR); categorical variables were summarised as frequencies and percentages. Because the count-based outcomes were non-normally distributed and strongly skewed in the reference group, between-group comparisons used the Mann–Whitney U test for continuous/ordinal variables and the chi-square or Fisher exact test for categorical variables; the Kruskal–Wallis test was used for three-group comparisons. Effect sizes were summarised with Cliff’s delta and with the prevalence ratio. Within-group bivariate associations were examined with Spearman’s rank correlation. A multivariable logistic regression model estimated the independent association between school context/social vulnerability and the presence of caries (global caries score > 0), adjusting for age, sex, toothbrushing frequency and sugar consumption; results are reported as adjusted odds ratios (ORs) with 95% CIs. Because caries prevalence was high in the vulnerable group, the adjusted OR would overestimate the corresponding relative risk; a modified Poisson regression model (log link; robust sandwich standard errors) using the same covariates was therefore fitted to estimate the adjusted prevalence ratio (PR), which is reported as the primary effect measure for the presence of caries, with the adjusted OR retained as a secondary measure. A sensitivity analysis re-fitted both models after excluding each escuela singular in turn to assess the influence of between-school heterogeneity. No formal a priori sample-size calculation was performed because no directly comparable within-setting effect size was available at the design stage; a post hoc calculation based on the observed proportions indicated that the achieved sample provided essentially complete power (>99%) to detect the observed difference in caries prevalence at α = 0.05. A supplementary multiple linear regression on the global caries score was performed for comparison.

### 2.7. Ethical Considerations

The study was conducted in accordance with the Declaration of Helsinki and approved by the Research Ethics Committee of the Catholic University of Valencia San Vicente Mártir (Project Code: UCV/2024-2025/101). Written informed consent was obtained from parents or legal guardians, and children participated under age-appropriate assent conditions. All data were anonymised and processed in compliance with the EU General Data Protection Regulation (GDPR).

## 3. Results

### 3.1. Characteristics of the Study Population

The vulnerable and reference groups were comparable in age and sex, with no significant differences in either variable (Table 1). This demographic similarity supports the interpretation that subsequent between-group differences were not primarily driven by age or sex composition.

### 3.2. Dental Caries Experience

**Figure 1:**
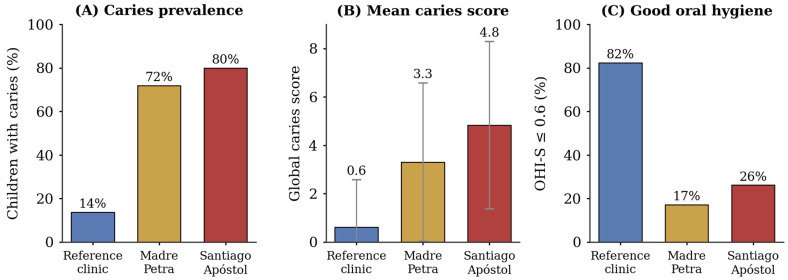
The oral health equity gap within a single setting. (**A**) Caries prevalence; (**B**) the mean global caries score (bars, mean, whiskers, SD); (**C**) the proportion of children with good oral hygiene (OHI-S ≤ 0.6), shown for the reference clinic group and for each escuela singular. A stepwise social gradient is evident across all three indicators.

**Figure 2:**
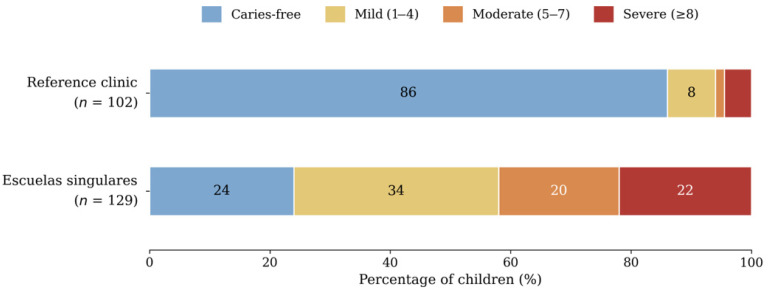
Distribution of caries severity by group. The reference clinic population is overwhelmingly caries-free, whereas children attending escuelas singulares are shifted towards moderate and severe caries experience, illustrating an almost inverted distribution.

Caries experience differed markedly between the groups (Table 2). A stepwise gradient across the two escuelas singulares and the reference clinic is shown in Figure 1: caries prevalence and mean caries score rose from the reference clinic (13.7%; 0.61 ± 1.96) to Colegio Madre Petra (71.9%; 3.30 ± 3.27) and Colegio Santiago Apóstol (80.0%; 4.83 ± 3.46), confirming that both escuelas singulares showed a substantially higher burden than the reference group, with some heterogeneity between them (see Section 4.4). The mean global caries score was 6.7 times higher in vulnerable children, and caries prevalence was 5.5 times higher. The contrast at the extremes of the distribution was also substantial: 86.3% of reference children were caries-free, compared with only 24.0% of vulnerable children, whereas severe caries experience (score ≥ 8) affected 21.7% of vulnerable children versus 3.9% of the reference group (Figure 2). The effect size was very large (Cliff’s delta = 0.64). Reported separately per WHO convention (Table 2), the permanent-dentition DMFT was 1.49 ± 1.87 in vulnerable children versus 0.21 ± 0.59 in reference children, and the primary-dentition dmft was 2.58 ± 3.06 versus 0.41 ± 1.55 (both *p* < 0.001; Mann–Whitney U test); both components therefore contributed to the global-score gap, supporting the use of the global caries score as a mixed-dentition summary measure alongside the WHO-standard DMFT and dmft indices.

### 3.3. Oral Hygiene Status

Oral hygiene was substantially poorer in vulnerable children (Table 3). While 82.4% of reference children had good hygiene and none reached the “poor” category, more than a third of vulnerable children (37.2%) were in the poor category. The effect size was very large (Cliff’s delta = 0.83).

### 3.4. Toothbrushing Frequency

Toothbrushing was less frequent in vulnerable children (Table 4). No reference child reported never brushing, whereas 9.3% of vulnerable children did; conversely, 79.4% of reference children brushed at least twice daily, compared with 50.4% of vulnerable children.

### 3.5. Sugar Consumption

Frequent sugar consumption was more common in vulnerable children: 64.1% reported higher-frequency intake versus 33.3% of reference children (Table 5).

### 3.6. Within-Group Correlation Analyses

Within both groups, the expected behavioural relationships were observed: poorer oral hygiene was associated with higher caries, and more frequent brushing with lower caries (Table 6). Sugar consumption was not directly correlated with the caries score in either group. The consistency of the hygiene and brushing associations indicates that behaviours operate in the same direction in both populations, even though the between-group gap is far larger than behavioural differences alone would predict. This null within-group correlation, together with the non-significant adjusted association for sugar in the multivariable model (OR 0.79, Table 7), appears to contradict the higher aggregate sugar-intake frequency reported by vulnerable children (Section 3.5) and should not be read as evidence that sugar plays a causal protective or neutral role. The most likely explanation is measurement imprecision: sugar was captured only as a simple frequency category using a non-validated instrument, which does not reflect the amount, timing or food matrix and is known to attenuate true associations towards the null; this limitation is noted explicitly here and in Section 4.6, and the discussion of dietary sugar throughout this manuscript should be read as descriptive rather than causal.

### 3.7. Multivariable Analysis

In the modified Poisson regression model, used as the primary multivariable model because of the high outcome prevalence, attendance at an escuela singular was associated with a substantially higher prevalence of caries after adjustment for age, sex, brushing frequency and sugar consumption (adjusted PR 5.39; 95% CI 3.25–8.92; *p* < 0.001). The logistic regression model, retained as a secondary analysis, yielded an adjusted OR of 20.50 (95% CI 9.60–43.76; *p* < 0.001), confirming the same direction of association but overestimating the relative effect because of the high prevalence of caries in the vulnerable group. More frequent brushing remained independently associated with lower odds of caries in the logistic model (OR 0.57 per category; 95% CI 0.36–0.91; *p* = 0.018), whereas age, sex and sugar consumption were not independently significant. A supplementary linear model for the global caries score yielded a consistent picture (vulnerability coefficient B = 3.33, *p* < 0.001; brushing B = −0.89, *p* < 0.001; model R^2^ = 0.33).

## 4. Discussion

### 4.1. Principal Findings

Within a single high-income city served by a formally universal health system, socially excluded children carried a caries burden that was several times greater than that of a contemporaneous reference paediatric population. The mean caries score was 6.7 times higher and caries prevalence was 5.5 times higher in vulnerable children, and the proportion of caries-free children was almost inverted between the groups (24.0% vs. 86.3%). After behavioural and demographic adjustment, attendance at an escuela singular remained associated with a more than fivefold higher prevalence of caries (adjusted PR 5.4), with a corresponding secondary adjusted OR of 20.5. Because both groups shared the same country, city, period and broad health-system context, the observed gap is unlikely to be explained solely by cross-system differences; nevertheless, the cross-sectional design supports association rather than causation.

### 4.2. A Steep Gradient Despite Universal Coverage

The most important implication of these results is that formal universal coverage does not necessarily guarantee equity in practice. Spain’s National Health System offers near-universal coverage, but oral health care remains only partly integrated into publicly funded services and access barriers may fall disproportionately on disadvantaged families [10,12]. The magnitude of the gap observed here is comparable to differences usually reported between high- and low-income countries [7,8], but it occurs entirely within a single high-income setting (underscoring that within-country social gradients can rival between-country differences) [5,6,19]. General-population caries levels in the Valencian region are far lower than those seen in our vulnerable group [11,13,14], so the excess is consistent with a strong social-exclusion gradient rather than with a high regional baseline.

### 4.3. Behaviours Matter, but Do Not Explain the Gap

Vulnerable children reported less frequent brushing and higher sugar-intake frequency. Within-group analyses supported the expected association for oral hygiene and brushing, but not for the simplified sugar-frequency variable. However, the behavioural differences were modest relative to the size of the outcome gap, and adjustment for brushing and sugar only partially addressed the association between vulnerability and caries (adjusted PR 5.4). This pattern indicates that the excess caries among socially excluded children is unlikely to be explained simply by “worse habits”; rather, it is consistent with the accumulated effects of social disadvantage, including constrained access to timely preventive and restorative care, lower oral-health literacy, material hardship and the social patterning of diet [3,15,20,21]. Interpreting the gap as a behavioural failing of families would therefore misattribute a structural problem to individual choice. This interpretation should nonetheless be tempered by measurement limitations: brushing and sugar were self-reported using a brief, non-validated instrument, so residual confounding by imprecisely captured behaviours cannot be excluded, and the persistence of the association after adjustment does not, by itself, prove that behavioural differences are irrelevant.

These findings align with broader oral-health equity research: individual behaviour operates within (and is bounded by) the structural and health-system context. In practice, better hygiene knowledge or motivation may not translate into lower caries when preventive services, restorative care, fluoride exposure, healthy food environments and continuity of follow-up are unevenly distributed [3,15,20,21]. Fluoride exposure deserves particular emphasis: neither water-fluoridation status nor fluoride toothpaste use/concentration was measured in this study, yet fluoride is among the most consistently protective factors against caries, and unmeasured differences in fluoride exposure between the two settings could plausibly contribute to part of the observed gap; this should be treated as an important unmeasured confounder rather than a factor that can be discounted.

### 4.4. Heterogeneity Between Vulnerable Schools

Even between the two escuelas singulares, caries experience and sugar consumption differed (mean caries 3.30 vs. 4.83; *p* = 0.013), illustrating that “vulnerability” is not monolithic and that local dietary and contextual factors modulate risk. This heterogeneity reinforces the value of school-level needs assessment when planning targeted interventions, rather than assuming a uniform risk profile across disadvantaged settings. A sensitivity analysis re-estimating the multivariable models after excluding each school in turn confirmed that the association was robust to this heterogeneity: the adjusted prevalence ratio ranged from 4.37 (95% CI 2.37–8.04) when Colegio Santiago Apóstol was excluded to 5.66 (95% CI 3.40–9.43) when Colegio Madre Petra was excluded (adjusted OR range 11.9–27.2), with both estimates remaining statistically significant and clinically large.

### 4.5. Implications for Policy and Practice

Given the cross-sectional design, these findings should be read as consistent with, rather than proof of the benefit of, a strategy of proportionate universalism: universal entitlement combined with additional, targeted resources for those with the greatest need [22]. Concretely, this means embedding school-based preventive programmes (supervised toothbrushing, fluoride varnish and fissure sealants, all with evidence of effectiveness) [23,24,25] together with mobile or outreach dental services and clear referral pathways into the universal system, with priority for escuelas singulares and similarly excluded communities. Population-level measures, such as context-appropriate water fluoridation where feasible and locally monitored [26], and policies to reduce free-sugar intake [27,28], complement these clinical actions. Integrating basic oral-health prevention into primary care and improving the affordability and reach of paediatric restorative care would address the access barriers that behavioural promotion alone cannot overcome. Behavioural support remains necessary (brushing was independently protective here), but it should be delivered as one component of a multilevel, equity-oriented approach consistent with the Common Risk Factor Approach [29] and with the WHO Global Oral Health Programme’s call to integrate oral health into wider health and social policy [30].

### 4.6. Strengths and Limitations

The principal strength is the within-setting design, which reduces confounding by country and broad health-system context; the standardised, calibrated clinical assessment and the multivariable adjustment further strengthen the comparison. Several limitations apply. The most important limitation concerns the reference group and residual confounding. The reference group comprised children attending university dental clinics; its caries-free proportion (86.3%) exceeds regional population-based estimates for the Valencia Community (around 63% at age 6 and 70% at age 12 based on deciduous/permanent prevalence, respectively [11]), indicating a likely positive-selection (healthy-user) bias and suggesting that the magnitude of the estimated gap should be regarded as an upper-bound, within-system estimate rather than a precise population parameter; it nonetheless remains a relevant non-excluded comparator from the same urban setting. Relatedly, individual-level socioeconomic indicators (parental education, household income, parental occupation, and migration background) were not collected, so attendance at an escuela singular captures a bundle of correlated socioeconomic disadvantage and its independent contribution cannot be fully separated from these unmeasured factors; residual confounding by individual-level SES is therefore likely and future work should incorporate direct SES measures. The cross-sectional design precludes causal inference, and the associational language used throughout this manuscript should be interpreted accordingly. Examiners were not blinded to examination setting, and setting-specific examination conditions (Section 2.5) may have contributed to differential detection, although this cannot be quantified with the available data. No participant flow diagram (numbers approached, declined or excluded) was recorded and no a priori sample-size calculation was performed, both of which are recommended by STROBE; a post hoc calculation (Section 2.6) indicated that the achieved sample was nonetheless adequately powered. Behavioural data were self-reported and subject to recall and social-desirability bias; sugar consumption was measured only as frequency using a non-validated instrument and did not capture the amount, timing, food matrix or total free-sugar exposure, which likely attenuated true associations (Section 3.6). OHI-S reflects a single time point. No radiographs were taken, potentially underestimating proximal caries. Structural variables such as service utilisation, fluoride exposure (Section 4.3) and previous preventive care were not directly measured and should be addressed in future longitudinal, mixed-methods research. Finally, although quality of life was not measured, the high burden of untreated and severe caries documented here is known to impair children’s daily functioning and wellbeing [21,31], underscoring the clinical relevance of the gap.

## 5. Conclusions

Within a single Spanish setting with formally universal health coverage, socially excluded children experienced a several-fold excess of dental caries compared with a contemporaneous reference paediatric population, together with poorer oral hygiene and less favourable behaviours. The excess persisted after adjustment for age, sex, brushing and a simplified sugar-frequency variable, suggesting that the observed gap is unlikely to be explained by the measured behavioural differences alone. These cross-sectional, within-setting findings suggest that formal coverage alone may not be sufficient to deliver oral health equity for the most disadvantaged children. Closing this gap may benefit from proportionate-universalism strategies that combine universal entitlement with targeted, school-based preventive and restorative care embedded within the public system, supported by dedicated financing mechanisms for high-need schools and outreach services, alongside continued behavioural support; longitudinal and multi-site studies are needed to confirm this association and to test such strategies directly.

## Figures and Tables

**Table 1:** Sociodemographic characteristics of the study population.

Characteristic	Escuelas Singulares (*n* = 129)	Reference Clinic (*n* = 102)	*p*-Value
Age, years (mean ± SD)	8.60 ± 1.81	8.69 ± 1.93	0.763 ^a^
Age, range	6–12	6–12	-
Male, n (%)	62 (48.1%)	50 (49.0%)	0.990 ^b^
Female, n (%)	67 (51.9%)	52 (51.0%)	

SD, standard deviation. ^a^ Mann–Whitney U test; ^b^ chi-square (χ^2^) test.

**Table 2:** Dental caries experience by study group.

Variable	Escuelas Singulares (*n* = 129)	Reference Clinic (*n* = 102)	*p*-Value
Global caries score, mean ± SD	4.07 ± 3.44	0.61 ± 1.96	<0.001 ^a^
DMFT (permanent teeth), mean ± SD	1.49 ± 1.87	0.21 ± 0.59	<0.001 ^a^
dmft (primary teeth), mean ± SD	2.58 ± 3.06	0.41 ± 1.55	<0.001 ^a^
Median (IQR)	4.00 (1.00–7.00)	0.00 (0.00–0.00)	
Range	0–11	0–10	
Caries prevalence (score > 0), %	76.0%	13.7%	<0.001 ^b^
Caries-free (score = 0), %	24.0%	86.3%	
Severe experience (score ≥ 8), %	21.7%	3.9%	

SD, standard deviation; IQR, interquartile range; ^a^ Mann–Whitney U test; ^b^ chi-square (χ^2^) test.

**Table 3:** Oral hygiene status (OHI-S) by study group.

Variable	Escuelas Singulares (*n* = 129)	Reference Clinic (*n* = 102)	*p*-Value
OHI-S, mean ± SD	1.49 ± 0.84	0.19 ± 0.34	<0.001 ^a^
Median (IQR)	1.60 (0.80–2.00)	0.00 (0.00–0.33)	
Good (0.0–0.6), n (%)	28 (21.7%)	84 (82.4%)	<0.001 ^b^
Fair (0.7–1.8), n (%)	53 (41.1%)	18 (17.6%)	
Poor (1.9–3.0), n (%)	48 (37.2%)	0 (0.0%)	

SD, standard deviation; IQR, interquartile range; ^a^ Mann–Whitney U test (mean OHI-S); ^b^ chi-square (χ^2^) test (category distribution).

**Table 4:** Toothbrushing frequency by study group.

Brushing Frequency	Escuelas Singulares (*n* = 129)	Reference Clinic (*n* = 102)	*p*-Value
Mean ± SD	1.57 ± 0.86	1.85 ± 0.50	0.002 ^a^
Never (0), n (%)	12 (9.3%)	0 (0.0%)	<0.001 ^b^
Once daily (1), n (%)	52 (40.3%)	21 (20.6%)	
Twice daily (2), n (%)	45 (34.9%)	75 (73.5%)	
Three+ daily (3), n (%)	20 (15.5%)	6 (5.9%)	

SD, standard deviation; ^a^ Mann–Whitney U test (mean frequency); ^b^ chi-square (χ^2^) test (distribution).

**Table 5:** Sugar consumption by study group.

Variable	Escuelas Singulares (*n* = 129)	Reference Clinic (*n* = 102)	*p*-Value
Mean ± SD	1.64 ± 0.48	1.33 ± 0.47	<0.001 ^a^
Higher-frequency intake (>1/day), %	64.1%	33.3%	<0.001 ^b^

SD, standard deviation; ^a^ Mann–Whitney U test; ^b^ chi-square (χ^2^) test.

**Table 6:** Spearman correlation coefficients between behavioural variables and the global caries score, within each group.

Variable Pair	Escuelas Singulares (ρ, *p*)	Reference Clinic (ρ, *p*)
OHI-S vs. caries score	0.207, 0.018	0.275, 0.005
Brushing vs. caries score	−0.221, 0.012	−0.246, 0.013
Sugar vs. caries score	−0.095, 0.285	−0.047, 0.637

ρ, Spearman’s rank correlation coefficient.

**Table 7:** Multivariable models predicting the presence of caries (global caries score > 0).

Variable	Effect Estimate (95% CI)	*p*-Value
Escuelas singulares vs. reference—adjusted PR, modified Poisson model	5.39 (3.25–8.92)	<0.001
Escuelas singulares vs. reference—adjusted OR, logistic model	20.50 (9.60–43.76)	<0.001
Age, per year—adjusted OR, logistic model	0.99 (0.82–1.19)	0.900
Female vs. male—adjusted OR, logistic model	1.74 (0.87–3.45)	0.116
Brushing, per category—adjusted OR, logistic model	0.57 (0.36–0.91)	0.018
Sugar, higher vs. lower—adjusted OR, logistic model	0.79 (0.38–1.63)	0.517

PR, prevalence ratio; OR, odds ratio; CI, confidence interval. The adjusted PR was estimated using modified Poisson regression with robust standard errors and is reported as the primary effect measure for the exposure variable. Adjusted ORs were estimated using logistic regression and are reported as secondary measures. Outcome: presence of caries (global caries score > 0). *n* = 231.

## Data Availability

The raw data supporting the conclusions of this article will be made available by the authors on request.

## Abbreviations

The following abbreviations are used in this manuscript:

DMFT	Decayed, missing due to caries and filled teeth in permanent dentition
dmft	Decayed, missing due to caries and filled teeth in primary dentition
GDPR	General Data Protection Regulation
IQR	Interquartile range
OHI-S	Simplified Oral Hygiene Index
OR	Odds ratio
PR	Prevalence ratio
SD	Standard deviation
SPSS	Statistical Package for the Social Sciences
STROBE	Strengthening the Reporting of Observational Studies in Epidemiology
WHO	World Health Organization
